# Aqueous and Methanolic Extracts of *Caulerpa mexicana* Suppress Cell Migration and Ear Edema Induced by Inflammatory Agents

**DOI:** 10.3390/md9081332

**Published:** 2011-08-10

**Authors:** Mariana Angelica Oliveira Bitencourt, Gracielle Rodrigues Dantas, Daysianne Pereira Lira, Jose Maria Barbosa-Filho, George Emmanuel Cavalcanti de Miranda, Barbara Viviana de Oliveira Santos, Janeusa Trindade Souto

**Affiliations:** 1 Department of Microbiology and Parasitology, Federal University of Rio Grande do Norte, Avenida Salgado Filho, BR 101, University Campus, Lagoa Nova, 59078-900, Natal, RN, Brazil; E-Mails: marianaobitencourt@yahoo.com.br (M.A.O.B.); graci_ufrn@hotmail.com (G.R.D.); 2 Laboratory of Technology Pharmaceutical, Federal University of Paraiba, 58051-900, Joao Pessoa, PB, Brazil; E-Mails: daysianneplira@yahoo.com.br (D.P.L.); jbarbosa@ltf.ufpb.br (J.M.B.-F.); 3 Laboratory of Marine Algae, Department of Systematics and Ecology, Federal University of Paraiba, 58051-900, Joao Pessoa, PB, Brazil; E-Mail: mirandag@dse.ufpb.br

**Keywords:** *Caulerpa mexicana*, green algae, zymosan, inflammation, edema, cell migration

## Abstract

The regulation of the inflammatory response is essential to maintaining homeostasis. Several studies have investigated new drugs that may contribute to avoiding or minimizing excessive inflammatory process. The aim of this study was to evaluate the effect of extracts of green algae *Caulerpa mexicana* on models inflammation. In mice, the inflammatory peritonitis model is induced by zymosan. Previous treatment of mice with aqueous and methanolic extracts of *C. mexicana* was able to suppress the cell migration to the peritoneal cavity, in a time-dependent but not in a dose-dependent manner. The treatment of mice with *C. mexicana* extracts also decreased the xylene-induced ear edema, exerting strong inhibitory leukocyte migration elicited by zymosan into the air pouch. We concluded that administration of the extracts resulted in a reduction of cell migration to different sites as well as a decrease in edema formation induced by chemical irritants. This study demonstrates for the first time the anti-inflammatory effect of aqueous and methanolic extracts from the green marine algae *Caulerpa mexicana*.

## 1. Introduction

The plant kingdom is responsible for the largest share of chemical diversity recorded to date in the literature and has contributed significantly to the research and discovery of new drugs of natural origin, as well as the supply of useful substances for treating diseases that affect living beings [1–12]. Natural products of marine origin have recently been the object of intense interest in research because of their potential pharmacological activities [13]. Marine algae are responsible for creating these natural products, producing a number of different compounds [14–16] with a range of pharmacological activities [17–23].

*Caulerpa mexicana* is a member of marine green alga Order Bryopsidales and the family Cauleparceae, which can be found in the temperate seas, but especially in tropical areas, such as the Brazilian Coastland [24]. These macrophytes are a source of several bioactive compounds, such as polysaccharides, terpenes and indole alkaloides, which exhibit different pharmacological activities such as antitumor, antiviral, antioxidant and anticoagulant effects [25–27]. They also display antibiotic, cytotoxic and antimitotic activity [28] and some studies have reported that marine algae products have anti-inflammatory properties [27,29].

The inflammatory response consists of a wide variety of physiological and pathological processes. A key feature of the inflammatory response is leukocytes sequestration from the blood to the tissues, which occurs in a sequential process. Initially, locally produced inflammation mediators, such as, TNF, IL-1, and IL-6, activate the vascular endothelium, which starts to express adhesion molecules that mediate tethering, rolling and cell adhesion. Polymorphonuclear cells and macrophages reach the tissues, migrate towards the inflammatory stimuli due to the chemokine gradient for phagocytes and eliminate the target [30]. An excessive inflammatory response can cause tissue damage. A well-regulated inflammatory process can protect the host against infections and remodel tissue structure, thereby re-establishing tissue function after injury. Acute inflammatory response, such as sepsis, can result in patient death, while chronic inflammations, such as asthma or autoimmune diseases, can cause irreversible tissue damage or induce organ failure [31,32]. Interestingly, there are no marine-derived anti-inflammatory natural products in clinical development at this time [33].

The regulation of inflammatory response is essential to maintain homeostasis, and several studies have investigated new drugs that may help avoid or minimize tissue injury caused by excessive inflammatory processes. The aim of this study was to evaluate the effect of extracts from the green alga *Caulerpa mexicana* in pro-inflammatory cytokines production by peritoneal macrophages *in vitro*, peritonitis, ear edema and air pouch inflammation *in vivo*. This is the first study to investigate the anti-inflammatory effect of the *Caulerpa mexicana* extracts in these inflammation models.

## 2. Results

### 2.1. The Effect of *Caulerpa mexicana* Extracts *in vitro* Production of Pro-Inflammatory Cytokines by Peritoneal Macrophages

**Figure 1.**
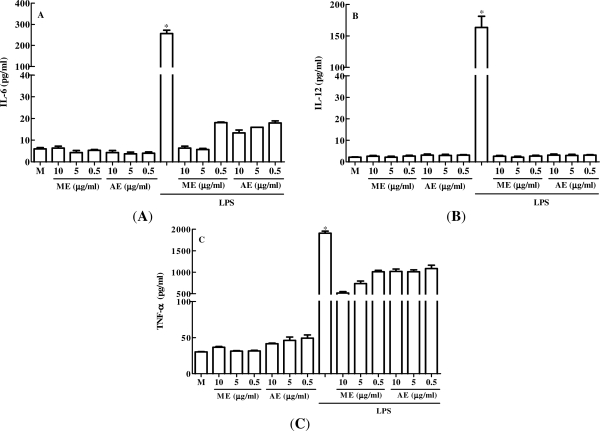
Effect of methanolic (ME) and aqueous (AE) extracts of *C. mexicana* on the production of IL-6 (**A**), IL-12 (**B**) and TNF-α (**C**). Peritoneal macrophages were cultured with 10, 5 and 0.5 μg/mL of *C. mexicana* extracts for 24 h with and without the presence of LPS (100 ng/mL). * *P* ≤ 0.005, compared to medium (M) and extract-treated groups.

First, we evaluated the *in vitro* anti-inflammatory effect of *C. mexicana* extracts, by determining whether these extracts could stimulate *in vitro* production of pro-inflammatory cytokines by peritoneal macrophages. Macrophages were cultured with different doses (10, 5, and 0.5 μg/mL) of the extracts for 24 h, both with and without the presence of lipopolysaccharide (LPS) from the cell wall of Gram-negative bacteria, which is known to stimulate the pro-inflammatory cytokines by these cells. As with cell cultured with only culture medium (M), methanolic (ME) and aqueous (AE) extracts of *C. mexicana* alone did not stimulate IL-6 (Figure 1A), IL-12 (Figure 1B) or TNF-α (Figure 1C) production by peritoneal macrophages. As was expected, LPS was able to stimulate intense production of these cytokines. Interestingly, when peritoneal macrophages were cultured with the extracts in the presence of LPS there was decreased production of IL-6, IL-12 and TNF. The data suggest that the extracts used in this study are unable to activate macrophages to produce pro-inflammatory cytokines and exhibit anti-inflammatory activity by inhibiting the production of the cytokines tested in the presence of LPS.

### 2.2. The Effect of Different Doses of *Caulerpa mexicana* Extracts on Zymosan-Induced Peritonitis in Mice

**Figure 2.**
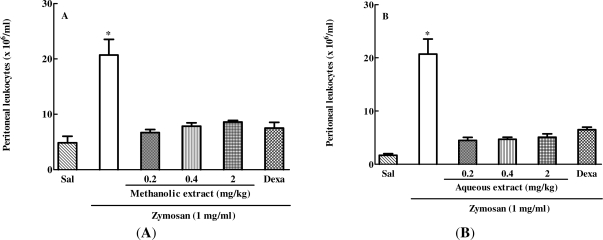
Effect of methanolic and aqueous extracts of *C. mexicana* zymosa-induced peritonitis model. Swiss mice were treated intravenously (i.v.) with methanolic (**A**) or aqueous (**B**) extract at doses of 2, 0.4, and 0.2 mg/kg or intraperitoneally (i.p.) with dexamethasone (0.5 mg/kg) and 30 min later were injected i.p. with 1 mL of zymosan (1 mg/mL). After six hours, peritoneal lavage was performed with saline and the cell number determined in a Neubauer chamber. * *P* ≤ 0.0001, compared to saline and drug-treated groups.

Next, the *in vivo* anti-inflammatory effect of aqueous and methanolic extracts of *C. mexicana* was evaluated in a zymosan-induced peritonitis model. As expected, the animals treated intravenously (i.v.) with saline and 30 min later intraperitoneally (i.p.) with zymosan showed intense leukocyte migration to the peritoneal cavity. On the other hand, groups treated with three different doses (2, 0.4 or 0.2 mg/kg) of methanolic (Figure 2A) or aqueous extracts (Figure 2B) showed a significant inhibition of cell migration to the peritoneal cavity when compared with the group administered saline i.v. and zymosan i.p. A reference drug, dexamethasone (DEXA), a commercially available steroidal anti-inflammatory drug, was used for greater data reliability. As expected, DEXA treatment significantly inhibited cell migration to the peritoneum of animals administered with zymosan. Therefore, when compared with the positive control (ZYM group), the drugs-treated groups (DEXA, ME and AE) exhibited anti-inflammatory activity, demonstrating impairment of leukocyte migration. However the effect of extracts was not dose-dependent, since the three doses tested induced similar inhibition of cell migration to the peritoneal cavity.

### 2.3. The Effect of *Caulerpa mexicana* Extracts on the Kinetics of Zymosan-Induced Peritonitis in Mice

**Figure 3.**
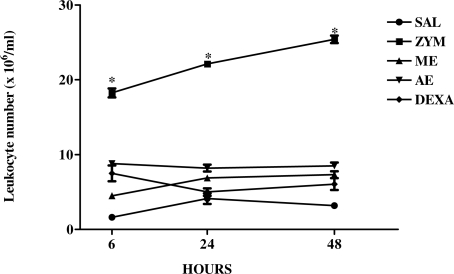
Kinetics of effect of aqueous and methanolic extracts of *C. mexicana* on zymosan-induced peritonitis. Swiss mice were treated intravenously with methanolic (ME) and aqueous (AE) (2 mg/kg) or intraperitoneally with dexamethasone (DEXA) at a dose of 0.5 mg/kg. Thirty minutes later they were injected intraperitoneally with 1 mL of zymosan (1 mg/mL). Peritoneal wash with saline was performed at 6, 24 and 48 h and the cell number determined in Neubauer chamber. *** *P* ≤ 0.0001, compared to saline and drug-treated groups.

After evaluating the effect of three different doses of *C. mexicana* extracts on zymosan-induced peritonitis, we then evaluated the effects of these extracts in the kinetics (6, 24 and 48 h) of zymosan-induced peritoneal cell migration using one single dose of the extracts. The results presented in Figure 3 show that without extract or DEXA treatment, zymosan induced a marked increase in the peritoneal cell migration in the three times analyzed (6, 24 and 48 h). However the previous treatment with the methanolic and aqueous extracts of *C. mexicana* (2 mg/kg) significantly inhibited the leukocyte migration to the peritoneal cavity during the time points studied, similar to that for dexamethasone. Extracts used in this study were therefore able to inhibit leukocyte migration to the peritoneal cavity even 48 h after zymosan inoculation.

### 2.4. The Effect of Extracts of *Caulerpa mexicana* on Xylene-Induced Ear Edema

After evaluating the effect of extracts of *C. mexicana* on the cell migration, we analyzed the effect of these extracts on another signal of inflammation: edema formation. The activity of methanolic and aqueous extracts of *C. mexicana* against xylene-induced ear edema is presented in the Table 1. Treating mice with 2 and 0.4 mg/kg doses of methanolic extract suppressed xylene-induced ear edema, reducing swelling by 73.55% and 77.07% after 15 min and by 83.75% and 85.63% after 1 h, respectively compared to the vehicle-treated group. On the other hand, when aqueous extract activity was analyzed, it was observed that the dose of 0.4 mg/kg reduced ear edema by 84.83% after 15 min and by 93.18% after 1 h, while the 2 mg/kg dose showed 25% and 36.88% ear edema inhibition 15 min and 1 h after analysis, respectively. Dexamethasone administration showed a decrease of 70.60% after 15 min and 73.37% 1 h after ear edema induction.

### 2.5. Effect of *Caulerpa mexicana* Extracts on the Zymosan-Injected Mouse Air Pouch

**Figure 4.**
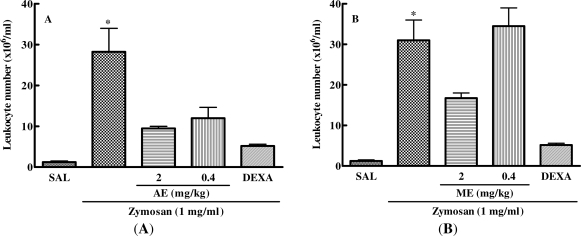
Effect of aqueous and methanolic extracts of *C. mexicana* on the leukocytes migration into the air pouch model. The animals were treated with saline or two different doses of aqueous (AE) or methanolic (ME) extract or with 0.5 mg/kg of dexamethazone (DEXA) and 30 min later receive 1 mL of zymosan solution (1 mg/mL) in the air pouch. Leukocyte migration was evaluated 24 h after zymosan injection into the air pouch. (**A**) * *P* ≤ 0.001, compared to saline and drug-treated groups; (**B**) * *P* ≤ 0.05, compared to saline, ME (2 mg/kg) and DEXA groups.

Zymosan injected into a mouse air pouch induced an acute inflammatory response characterized by plasma exudation and leukocyte infiltration to the cavity. This demonstrated that previous treatment of mice with the two different doses of the aqueous extract of *C. mexicana* or with dexamethasone significantly decreased the pro-inflammatory effect of zymosan, thus reducing the cell migration to the inflammation site. However, methanolic extract was able to decrease the zymosan-induced cell migration into the air pouch only at a dose of 2 mg/kg (Figure 4).

**Figure 5.**
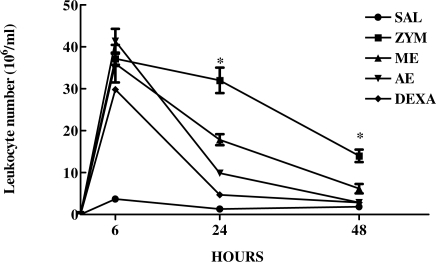
Effect of methanolic and aqueous extracts of *C. mexicana* on the kinetic of cell migration into the air pouch. The animals were treated with saline (SAL), 2 mg/mL of the aqueous (AE) or methanolic (ME) extract or with 0.5 mg/kg of dexamethazone (DEXA) and 30 min later received 1 mL of zymosan solution (1 mg/mL) in their air pouch. At the time points indicated, leukocyte number was determined after zymosan injection into the air pouch. * *P* ≤ 0.05, compared to saline and drug-treated groups.

After analyzing the dose response of aqueous and methanolic extracts of *C. mexicana* on zymosan-induced leukocyte accumulation into the mouse air pouch, we evaluated the kinetics of this response (6, 24 and 48 h) using a fixed dose of these extracts (2 mg/kg). Figure 5 shows that the aqueous and methanolic extract of *C. mexicana* significantly decreased cell migration to the dorsal cavity at 24 and 48 h after zymosan injection. Inhibition of cell migration by the extracts was similar to that one induced by dexamethasone, which inhibited cell migration to the air pouch cavity at the three time points analyzed.

## 3. Discussion

In this study we have examined the anti-inflammatory effect of aqueous and methanolic extracts of *Caulerpa mexicana*, analyzing their effects on *in vitro* pro-inflammatory and immune stimulatory cytokine production by peritoneal macrophages, as well as on different *in vivo* inflammation models, such as peritonitis and zymosan-induced air pouch cell migration and xylene-induced ear edema.

Macrophages activation is an important cell in immune and inflammatory response, which can start after microbial products interact with pattern recognition receptors (PRR) present in the macrophages’ membranes. LPS from the cell wall of Gram negative bacteria is a classical example of a microbial product that can activate macrophages through PRR and induce pro-inflammatory cytokine production, such as IL-6, IL-1 and TNF, as well as immunostimulatory cytokine, as IL-12 [34]. To determine if the extracts of *C. mexicana* could have any stimulatory activity in macrophages, we cultured these cells with different doses of the methanolic and aqueous extracts for 24 h, with and without the presence of LPS. The results are interesting because extracts alone did not stimulate pro-inflammatory cytokine production and were able to inhibit production of these LPS-induced cytokines, exhibiting *in vitro* anti-inflammatory activity. Moreover, mechanisms of action of these extracts remain unknown, one possibility being that they contain substances that inhibit or compete [35] with the LPS ligand, the Toll-like receptor 4 (TLR4) or CD14 molecule, which is a co-receptor for LPS [36], or bind with some molecules on the macrophage surface, inhibiting LPS-induced biochemical signals for pro-inflammatory cytokine production [37]. Further investigations will focus on the chemical identification and mechanisms of action of the major active components responsible for anti-inflammatory activity in *C. mexicana* extracts.

As we did not observe *in vitro* macrophage activation induced by the extracts of *C. mexicana*, the next step was to determine if they could have any anti-inflammatory activity in *in vivo* inflammatory models. Peritonitis is a serious infection that often leads to multiple organ failure, septicemia, and mortality [38]. Acute inflammatory reactions can be induced experimentally by a variety of substances. Zymosan, the insoluble polysaccharide component of the *Saccharomyces cerevisiae* cell wall is commonly used to induce acute peritonitis in mice [39]. This study showed that the aqueous and methanolic extracts exerted an anti-inflammatory effect, reducing zymosan-induced cell recruitment into the peritoneal cavity. These data are consistent with the results of the studies demonstrating that extracts of alga *C. racemosa* as well as purified alkaloids from the genus *Caulerpa* showed anti-inflammatory and analgesic activity [40,41]. The process by which the extracts used in this study inhibit cell migration in response to inflammatory stimuli has yet to be elucidated. One possibility is that these extracts contain substances that act in an inhibitory or competitive fashion with zymosan, interacting with one of the zymosan’s receptors in the cell surfaces, the Toll like receptor 2 (TLR2) [42], blocking cell adhesion to vascular endothelium [43] or inhibiting the macrophages’ activation via scavenger receptors [44]. We did not detect any statistical difference in pharmacological action between the doses of aqueous and methanolic extracts tested in this model. We believe that this occurred because dose concentration was very similar and may therefore be in the therapeutic window, because when a previous experiment was conducted using a dose of 20 mg/kg, there was no anti-inflammatory activity (data not shown). Another important point to consider is that when some pharmacological preparations are made from one plant extract, different solvents are used as carrier, thereby obtaining the highest possible number of components. The absence of a difference in anti-inflammatory activity when methanolic or aqueous extracts were used may be due to the solvents used (water and methanol), which, in addition to being chemically different, may have extracted similar components that are responsible for the anti-inflammatory activity observed in our models. In some Caulerpa species, for example *C. racemosa*, purified indole alkaloids, such as caulerpin, have proven anti-inflammatory activity [41]. Therefore, new studies are needed to isolate the extract compounds and evaluate their mechanisms of action.

Xylene-induced ear edema was adopted as the acute inflammatory model, since it has frequently been used to evaluate the anti-inflammatory effects of natural products and has good predictive value in the screening anti-inflammatory agents [45,46]. Xylene-induced ear edema in this study led to fluid accumulation and edema, characteristic of the acute inflammatory response. The xylene-induced inflammation caused the release of proinflammatory mediators from sensory neurons that act on peripheral target cells, such as mast cells and other immune cells, producing neurogenic inflammation characterized by warmth, redness and edema [47]. Suppression of this response is a likely indication of antiphlogistic effect [46] and the results clearly showed that methanolic and aqueous extracts of *C. mexicana* exerted significant antiphlogistic effects against xylene-induced ear edema. These data are consistent with the studies showing that plant extracts from traditional Tibetan and Chinese medicine [45,46] and seaweed extracts [48] presented significant reduction in the ear edema in mice.

The local zymosan-induced inflammatory response in the air pouch in mice is considered similar to the inflammatory response of synovial tissue [48] due to injection of sterile air into the backs of mice, forming a cell-lined cavity resembling the synovial membrane [49,50]. Therefore, when injected into the cavity, zymosan is phagocytosed, inducing degranulation and neutrophil respiratory burst, and production of several inflammatory mediators such as myeloperoxidase, eicosanoids, phospholipase A2, nitric oxide, tumor necrosis factor-α (TNF-α) and interleukin 1 (IL-1) and IL-6 [50]. This model was used to investigate the anti-inflammatory effect of aqueous and methanolic extracts of *C. mexicana*, measuring the number of leukocytes migration to the air pouch. The extracts tested in our experiments, proved to have anti-inflammatory agents, similarly to those observed in [51,52], leading us to speculate on the substance contained in these extracts that exert an anti-inflammatory activity and their mechanism of action, issues of interest for future study. The mechanism used by the methanolic and aqueous extract in this inflammation model is still unknown, but we suggest that it may be due to endothelium receptor binding, thereby preventing leukocyte rolling into the dorsal cavity, as well as inhibiting the production of cytokines responsible for triggering the inflammatory process [42,44]. Interestingly, when we compared experimental ear edema and air pouch inflammation models, we observed that a 2 mg/kg dose of aqueous extract was much more effective in inhibiting cell migration to the air pouch model than reducing xylene-induced edema formation in the ear. We believe that this difference may have occurred because of the different experimental models used, possibly exhibiting different mechanisms of action, or because of different administration routes. Since the bioactive compound responsible for biological activity has yet to be identified and the manner in which it operates in animal organisms is still unknown, it is suggested that future trials involve bioassay-guided fractionation of these extracts.

## 4. Experimental

### 4.1. Extraction and Isolation

The alga *C. mexicana* was collected in the coastal region of Bessa (7°03′52″S/34°49′51″W), Joao Pessoa, Paraiba State, Brazil in April 2008. The specimen was identified by Dr. George Emmanuel Cavalcanti de Miranda. Voucher specimens of *C. mexicana* (JPB 13985) have been deposited in the Lauro Pires Xavier Herbarium at the Federal University of Paraiba, Brazil. Fresh algae were lyophilized and exhaustively extracted with methanol and water in a Soxhlet apparatus, to obtain the respective extracts.

### 4.2. Animal

Male Swiss albino and BALB/c mice (6–8 weeks old) were used in the experiment. All mice were housed, 5–6 per cage at a room temperature of 22 ± 2 °C, and a 12 h:12 h light/dark cycle. They had free access to food and water. Groups of five animals were used in each test group and control animals received saline only. All *in vivo* experiments were approved by the local ethics committee.

### 4.3. Peritoneal Macrophage Cell Culture

BALB/c mice were inoculated with 1 mL of sterile 3% sodium thioglycollate (Sigma). After three days the animals were killed and macrophages harvested by washing their peritoneal cavity with 5 mL of cold 0.9% w/v sterile saline. The cell suspension was centrifuged at 250× *g* for 10 min at 4 °C, the supernatant discarded, the cell bottom dissolved in 1 mL of incomplete medium and cell number determined using a Neubauer chamber. One ml of cell suspension (1 × 10^6^/mL) was incubated for 2 h for the macrophage adhesion to the 24-well cell culture plate. Non-adherent cells were harvested by three repeat washing with 1 mL of cell culture medium. Adherent cells were incubated with different doses of methanolic and aqueous extracts of *C. mexicana* (50, 10 and 5 μg/mL) and one hour after the cells were incubated with the extracts, LPS (100 ng/mL) was added to one group. Macrophages were also incubated only with medium or LPS as a negative and positive control, respectively. After 24 h of cell culture, supernatants were collected for determination of IL-6, IL-12 and TNF-α levels, which was performed using an enzyme-linked immunosorbent assay kit from Ebioscience.

### 4.4. Induction of Peritonitis

Peritoneal inflammation was induced according to the procedure described previously in [39] with a few modifications. Animals were inoculated intravenously (i.v.) with saline, methanolic or aqueous extracts (2, 0.4, or 0.2 mg/kg) of *C. mexicana* or intraperitonealy with dexamethasone (0.5 mg/kg). Thirty minutes later, the animals were injected intraperitoneally with 0.9% w/v sterile saline or zymosan A (Sigma), freshly prepared (1 mL of 1% w/v) in sterile saline. At the selected time points (6, 24, and 48 h), the animals were killed by cervical dislocation, and peritoneal exudates harvested by peritoneal wash with 5 mL of cold saline. Exudates were centrifuged at 250× *g* for 10 min, at 4 °C, and the total cells number determinate in hemocytometer following staining with Turk’s solution.

### 4.5. Xylene-Induced Ear Edema

The effects of extracts on acute topical inflammation were evaluated according [46]. The mice were treated intravenously with saline, methanolic or aqueous extracts of *C. mexicana* (2 mg/kg and 0.4 mg/kg) or intraperitonealy with 0.5 mg/kg of dexamethasone. Thirty minutes after the treatment, ear edema was induced by applying 0.04 mL of xylene (Merk) to the anterior and posterior surfaces of the right ear. The left ear was considered as control and only saline was applied. Fifteen minutes and one hour after xylene application, mice were killed and both ears were removed. Circular sections were taken, using a cork borer with a diameter of 7 mm, and weighed. Inhibition level (%) was calculated according to the following equation:

(1) Inhibition (%)=[1−Et/Ec]×100

where *Et* = average edema in the treated group, and *Ec* = average edema of the control group.

### 4.6. Mouse Air Pouch Model

The air pouch was produced in mice as previously described [49,53]. Briefly, 3 mL of sterile air was subcutaneously injected into the back of the experimental animals. Three days later 1.5 mL of sterile air was injected into the cavity. Six days after the initial air injection, animals were injected intravenously, with methanolic or aqueous extract (2 mg/kg, 0.4 mg/kg and 0.2 mg/kg) or intraperitoneally with dexamethasone (0.5 mg/kg). Thirty minutes later zymosan solution (1 mL of 1% w/v) was injected into the air pouch. At the selected time points (6, 24, and 48 h), animals were killed by cervical dislocation and exudates harvested from each air pouch by washing them out with 1 mL of 0.9% w/v sterile saline. The exudates collected from each mouse were centrifuged at 250× *g* for 10 min at 4 °C; the cell bottom was diluted in 1 mL of saline and the number of cells determined in hemacytometer.

## 5. Conclusions

In conclusion, this is the first study to evaluate the anti-inflammatory activity of extracts of *Caulerpa mexicana* using microbial products. *In vitro* data demonstrate that the extracts were unable to stimulate macrophages to produce IL-6, IL-12 and TNF-α, but were able to inhibit the production of these LPS-induced cytokines, showing *in vitro* anti-inflammatory activity. In *in vivo* studies using zymosan-induced peritonitis, air pouch inflammation and xylene-induced ear edema exhibited a significant anti-inflammatory effect. Further studies are required to determine the chemical composition of *C. mexicana* extracts and their possible anti-inflammatory mechanisms of action.

## Figures and Tables

**Table 1.** Anti-inflammatory effects of extracts on xylene-induced ear edema in mice.

**Groups**	**15 min**			**1 h**	

	**Dose (mg/kg)**	**Difference (mg)**	**Inhibition (%)**	**Difference (mg)**	**Inhibition (%)**
**Saline (i.v.)**	–	32.45 ± 2.603	–	31.80 ± 5.289	–
**Methanolic**	0.4	7.475 ± 2.809	77.07% **	4.567 ± 2.668	85.63% **
**Extract (s.c.)**	2	8.453 ± 3.500	73.55% **	5.167 ± 2.580	83.75% **
**Aqueous**	0.4	4.933 ± 4.916	84.86% ***	2.167 ± 3.316	93.18% **
**Extract (s.c.)**	2	24.45 ± 5.212	25%	20.07 ± 5.029	36.88%
**Dexa (i.p.)**	0.5	9.600 ± 2.419	70.60% **	8.467 ± 2.850	73.37% **

Values are mean ± standard deviation (S.D.), *n* = 5,

** *p* < 0.001,

*** *p* < 0.0001, compared to saline group.

## References

[1] Vuorella P, Leinonenb M, Saikkuc P, Tammelaa P, Rauhad JP, Wenneberge T, Vuorella H (2004). Natural products in the process of finding new drug candidates. Curr. Med. Chem.

[2] Itokawa H, Morris-Natschke SL, Akiyama T, Lee KH (2008). Plant-derived natural product research aimed at new drug discovery. J. Nat. Med.

[3] Falcão HS, Mariath IR, Diniz MFFM, Batista LM, Barbosa-Filho JM (2008). Plants of the American continent with antiulcer activity. Phytomedicine.

[4] Agra MF, Silva KN, Basílio IJLD, França PF, Barbosa-Filho JM (2008). Survey of medicinal plants used in the region Northeast of Brazil. Braz. J. Pharmacogn.

[5] Sousa FCF, Melo CTV, Citó MCO, Félix FHC, Vasconcelos SMM, Fonteles MMF, Barbosa Filho JM, Viana GSB (2008). Medicinal plants and their bioactive constituents: A scientific review of bioactivity and potential benefits in the anxiety disorders in animal models. Braz. J. Pharmacogn.

[6] Barbosa-Filho JM, Alencar AA, Nunes XP, Tomaz ACA, Sena-Filho JG, Athayde-Filho PF, Silva MS, Souza MFV, Cunha EVL (2008). Sources of alpha-, beta-, gamma-, delta- and epsilon-carotenes: a twentieth century review. Braz. J. Pharmacogn.

[7] Quintans-Júnior LJ, Almeida JRGS, Lima JT, Nunes XP, Siqueira JS, Oliveira LEG, Almeida RN, Athayde-Filho PF, Barbosa-Filho JM (2008). Plants with anticonvulsant properties—a review. Braz. J. Pharmacogn.

[8] Falcão HS, Leite JA, Barbosa-Filho JM, Athayde-Filho PF, Chaves MCO, Moura MD, Ferreira AL, Almeida ABA, Souza-Brito ARM, Diniz MFFM (2008). Gastric and duodenal antiulcer activity of alkaloids: A review. Molecules.

[9] Mota KSL, Dias GEN, Pinto MEF, Luiz-Ferreira A, Souza-Brito ARM, Hiruma-Lima CA, Barbosa-Filho JM, Batista LM (2009). Flavonoids with gastroprotective activity. Molecules.

[10] Mariath IR, Falcão HS, Barbosa-Filho JM, Sousa LCF, Tomaz ACA, Batista LM, Diniz MFFM, Athayde-Filho PF, Tavares JF, Silva MS (2009). Plants of the American continent with antimalarial activity. Braz. J. Pharmacogn.

[11] Ribeiro-Filho J, Falcão HS, Batista LM, Barbosa-Filho JM, Piuvezam MR (2010). Effects of plant extracts on HIV-1 protease. Curr. HIV Res.

[12] Silva FL, Fischer DCH, Tavares JF, Silva MS, Athayde-Filho PF, Barbosa-Filho JM (2011). Compilation of Secondary Metabolites from *Bidens pilosa* L. Molecules.

[13] Mayer AMS, Rodriguez AD, Berlinck D, Fusetani N (2011). Marine pharmacology in 2007–2008: Marine compounds with antibacterial, anticoagulant, antifungal, anti-inflammatory, antiprotozoal, antituberculosis and antiviral activities; affecting the immune and nervous system, and other miscellaneous mechanisms of action. Comp. Biochem. Physiol. C.

[14] Cen-Pacheco F, Nordstrom L, Souto ML, Martin MN, Fernandez JJ, Daranas AH (2010). Studies on polyethers produced by red algae. Mar. Drugs.

[15] Guven KC, Percot A, Sezik E (2010). Alkaloids in marine algae. Mar. Drugs.

[16] Cabrita MT, Vale C, Rauter AP (2010). Halogenated compounds from marine algae. Mar. Drugs.

[17] Genovese G, Tedone L, Hamann MT, Morabito M (2009). The mediterranean red alga *Asparagopsis*: A source of compounds against *Leishmania*. Mar. Drugs.

[18] O'Sullivan L, Murphy B, McLoughlin P, Duggan P, Lawlor PG, Hughes H, Gardiner GE (2010). Prebiotics from marine macroalgae for human and animal health applications. Mar. Drugs.

[19] Pallela R, Na-Young Y, Kim SK (2010). Anti-photoaging and photoprotective compounds derived from marine organisms. Mar. Drugs.

[20] Smith VJ, Desbois AP, Dyrynda EA (2010). Conventional and unconventional antimicrobials from fish, marine invertebrates and micro-algae. Mar. Drugs.

[21] Cantillo-Ciau Z, Moo-Puc R, Quijano L, Freile-Pelegrin Y (2010). The tropical brown alga *Lobophora variegata*: A source of antiprotozoal compounds. Mar. Drugs.

[22] Iannitti T, Palmieri B (2010). An update on the therapeutic role of alkylglycerols. Mar. Drugs.

[23] Zhang JL, Xia WS, Liu P, Cheng QY, Tahirou T, Gu WX, Li B (2010). Chitosan modification and pharmaceutical/biomedical applications. Mar. Drugs.

[24] Neto JTBB, Rodrigues JAG, Pontes GC, Farias WRL (2008). Sulfated polysaccharides of the alga *Caulerpa sertularioides* (GMEL.) Howe: Analysis of methods of precipitation. Rev. Bras. Eng. Pesca.

[25] Shen WZ, Wang H, Guo GQ, Tuo JJ (2008). Immunomodulatory effects of *Caulerpa racemosa* var *peltata* polysaccharide and its selenizing product on T lymphocytes and NK cells in mice. Sci. China Ser. C.

[26] Torres MR, Sousa APA, Silva Filho EAT, Pessoa CO, Moraes MEA, Moraes MO, Lotufo LVC (2005). Biological activity of aqueous and organic extracts of seaweeds from Ceará state, Brazil. Arq. Cien. Mar..

[27] Rocha FD, Pereira RC, Kaplan MAC, Teixeira VL (2007). Produtos naturais de algas marinhas e seu potencial antioxidante. Braz. J. Pharmacogn.

[28] Ballesteros E, Martins D, Uriz MJ (1992). Biological activity of extracts from some Mediterranean Macrophytes. Bot. Mar.

[29] Cumashi A, Ushakova NA, Preobrazhenskaya ME, D’Incecco A, Piccoli A, Totani L, Tinari N, Morozevich GE, Berman AE, Bilan MI (2007). A comparative study of the anti-inflammatory, anticoagulant, antiangiogenic, and antiadhesive activities of nine different fucoidans from brown seaweeds. Glycobiology.

[30] Luster AD (1998). Chemokines-chemotactic cytokines that mediate inflammation. N. Engl. J. Med.

[31] Castellheim A, Brekke OL, Espevik T, Harboe M, Mollnes TE (2009). Innate immune responses to danger signals in systemic inflammatory response syndrome and sepsis. Scand. J. Immunol.

[32] Shin DQ, Targan SR (2008). Immmunopathogeneses of inflammatory bowel disease. World J. Gastroenterol.

[33] Mayer AMS, Glaser KB, Cuevas C, Jacobs RS, Kem W, Little RD, McIntosh JM, Newman DJ, Potts BC, Shuster DE (2010). The odyssey of marine pharmaceuticals: A current pipeline perspective. Trends Pharmacol. Sci.

[34] Takeuchi O, Akira S (2010). Pattern recognition receptors and inflammation. Cell.

[35] Yang EJ, Moon JY, Kim MJ, Kim DS, Kim CS, Lee WJ, Hyun CG (2010). Inhibition effect of Jeju endemic seaweeds on the production of pro-inflammatory mediators in mouse macrophage cell line Raw 264.7. J. Zhejiang. Univ. Sci. B.

[36] Mollnes TE, Christiansen D, Brekke OL, Espevik T (2008). Hypothesis: Combined inhibition of complement and CD14 as treatment regimen to attenuate the inflammatory response. Adv. Exp. Med. Biol.

[37] Kazlowska K, Hsu T, Hou CC, Yang WC, Tsai GJ (2010). Anti-inflammatory properties of phenolic compounds and crude extract from *Porphyra dentata*. J. Ethanophamacol.

[38] Kolaczkowska E, Seljelid R, Plytycz B (2001). Role of mast cells in zymosan-induced peritoneal inflammation in Balb/c and mast cell-deficient WBB6F1 mice. J. Leukoc. Biol.

[39] Kolaczkowska E, Koziol A, Plytycz B, Arnold B (2010). Inflammatory macrophages, and not only neutrophils, die by apoptosis during acute peritonitis. Immunobiology.

[40] Souza ET, Lira DP, Queiroz AC, Silva DJC, Aquino AB, Mella EA, Lorenzo VP, Miranda GEC, Araújo-Júnior JX, Chaves MCO (2009). The antinoceptive and anti-inflammatory activities of caulerpin, a bisindole alkaloid, isolated from seaweeds of the genus *Caulerpa*. Mar. Drugs.

[41] Souza ET, Queiroz AC, Miranda GEC, Lorenzo VP, Silva EF, Freire-Dias TLM, Cupertino-Silva YK, Melo GMA, Santos BVO, Chaves MCO (2009). Antinociceptive activities of crude methanolic extract and phases, *n*-butanolic, chloroformic and ethyl acetate from *Caulerpa racemosa* (Caulerpaceae). Braz. J. Pharmacogn.

[42] Kelly MM, Mcnagny K, Williams DL, Van Rooijen N, Maxwell L, Gwozd C, Mody CH, Kubes P (2008). The lung responds to zymosan in a unique manner independent of toll-like receptors. Am. J. Respir. Cell Mol. Biol.

[43] Ushakova NA, Preobrazhenskaya ME, Nifantiev NE, Usov AI, Pochechueva TV, Galanina OE, Bovin NV (1999). Inhibitory activity of monomeric and polymeric selectin ligands. Vopr. Med. Khim.

[44] Krieger M, Herz J (1994). Structures and functions of multiligand lipoprotein receptors: Macrophage scavenger receptors and LDL receptor-related protein (LRP). Annu. Rev. Biochem.

[45] Zhang Z, Luo P, Li J, Yi T, Wang J, An J, Zhang H (2008). Comparison of the anti-inflammatory activities of three medicinal plants known as “Meiduoluomi” in Tibetan folk medicine. Yakugaku Zassi.

[46] Parveen Z, Deng Y, Saeed MK, Dai R, Ahamad W, Yu YH (2007). Antiiflammatory and analgesic activities of *Theseus chinese* Turcz extracts and its major flavonoids, kaempferol and kaempferol-3-*O*-glucoside. Pharm. Soc. Jpn.

[47] Richardson JD, Vasko MR (2002). Cellular mechanisms of neurogenic inflammation. J. Pharmacol. Exp. Ther.

[48] Khan MN, Choi JS, Lee MC, Kim E, Nam TJ, Hong YK (2008). Anti-inflammatory activities of methanol extracts from various seaweed species. J. Environ. Biol.

[49] Yoon SY, Kwon YB, Kim HW, Roh DH, Kang SY, Kim CY, Han HJ, Kim KW, Yang IS, Beitz AJ, Lee JH (2005). Intrathecal neostigmine reduces the zymosan-induced inflammatory response in a mouse air pouch model via adrenomedullary activity: Involvement of spinal muscarinic type 2 receptors. Neuropharmacology.

[50] Cabrera PV, Blanco G, Gravisaco MJ, Alvarez E, Hajos S (2001). Zymosan modulates CD44 isoform expression in a murine model of inflammation resembling rheumatoid arthritis synovitis. J. Rheumatol.

[51] Kim EH, Shim B, Kang S, Jeong G, Lee LS, Yu YB, Chun M (2009). Anti-inflammatory effects of *Scutellaria baicalensis* extract via suppression of immune modulators and MAP kinase signaling molecules. J. Ethanopharmacol.

[52] Kang SY, Yoon SY, Roh DH, Jeon MJ, Seo HS, Uh DR, Kwon YB, Kim HW, Han HJ, Lee HJ (2008). The anti-arthritic effect of ursolic acid on zimosan-induced acute inflammation and adjuvant-induced chronic arthritis models. J. Pharm. Pharmacol.

[53] Vigil SVG, Liz R, Medeiros YS, Fröde TS (2008). Efficacy of tacrolimus in inhibiting inflammation caused by carrageenan in a murine model of air pouch. Transpl. Immunol.

